# Immune status of children with obstructive sleep apnea/hypopnea syndrome

**DOI:** 10.12669/pjms.331.11959

**Published:** 2017

**Authors:** Zihe Zhang, Chunguang Wang

**Affiliations:** 1 Zihe Zhang, Department of Otolaryngology, Qilu Children’s Hospital of Shandong University, Ji’nan 250022, Shandong Province, China; 2 Chunguang Wang, Department of Laboratory Medicine, Anqiu People’s Hospital, Weifang 262100, Shandong Province, China

**Keywords:** Children, Hypopnea syndrome, Immune status, Obstructive sleep apnea

## Abstract

**Objective:**

We aimed to evaluate the immune status of children with obstructive sleep apnea/hypopnea syndrome (OSAHS).

**Methods:**

Fifty children with OSAHS having the symptoms of “snoring, mouth breathing and suffocating during sleep”, who were admitted in our hospital from May 2014 to May 2016, were randomly selected. Another 52 healthy, age- and gender-matched children were enrolled as control subjects after taking informed consent. After admission, the peripheral venous blood was collected. T cell subsets and cytokines were analyzed by flow cytometry. Immunoglobulin and complement levels were detected by immunoassay analyzer.

**Results:**

The percentage of CD8^+^ T lymphocytes in children with OSAHS was (26.47 ± 1.52)% which was significantly higher than that of control group ((21.94 ± 1.92)%) (P<0.05). OSAHS group had a significantly lower CD4^+^/CD8^+^ ratio (1.24 ± 0.12) than that of control group (1.45 ± 0.11) (P<0.05). The two groups had similar percentages of CD3^+^ and CD4^+^ T lymphocytes (P>0.05). OSAHS group had significantly higher serum levels of IL-4, IL-6, IL-10 and IFN-γ than those of control group (P<0.05), but their IL-2 and TNF-α levels were similar (P>0.05). The serum IgA and C3 levels of OSAHS group significantly exceeded those of control group (P<0.05), but their IgG, IgM and C4 levels were similar (P>0.05).

**Conclusion:**

Children with OSAHS had increased percentage of CD8^+^ T lymphocytes and decreased CD4^+^/CD8^+^ ratio, suggesting this group had poor immune function. Increase in humoral immune-related indices IL-4, IL-6, IL-10 and IFN-γ indicated the occurrence of oxidative stress and systemic inflammatory status.

## INTRODUCTION

Obstructive sleep apnea/hypopnea syndrome (OSAHS), which is characterized by intermittent partial or complete obstruction of the upper airway during sleep, endangers from neonates to adolescents, with the overall morbidity rates of 1-4%.^1^,^2^ Due to multisystem damages induced by sleep respiratory flow changes, repeated hypoxemia, carbon dioxide retention and repeated awakening, children with OSAHS usually suffer from complications such as growth retardation, cardiac function changes, conductive deafness, facial deformity, memory loss, mental decline and personality changes.^3^ Children aged from 2-6 years old are mostly prone to OSAHS, being closely associated with the physiological hypertrophy of adenoids and tonsils.^4^ Most studies concerning OSAHS only detected humoral or cellular immune responses. It is thus of great significance to evaluate the immune functions of children with OSAHS in order to clarify the risks of this disease and to perform effective treatment. In this study, the effects of OSAHS on the humoral and cellular immunity of children were assessed by collecting peripheral venous blood samples and detecting T cell subsets, immunoglobulins, complements and cytokines.

## METHODS

From May 2014 to May 2016, 450 children with OSAHS having the symptoms of “snoring, mouth breathing and suffocating during sleep” admitted in our hospital were numbered, from whom 50 children were selected by using the random number method. This study has been approved by the ethics committee of our hospital. Written consent was obtained from the guardians of all enrolled children. The patients consisted of 40 boys and 10 girls, with the mean age of 79.38 months (47-148 months) and the disease courses from three months to 10 years. All patients were diagnosed as OSAHS by all-night sleep monitoring after admission with the following indices: apnea-hypopnea index (AHI) of 1.2-98.2, obstructive apnea index of 1-88.3 and minimum blood oxygen saturation <92%. All children with OSAHS were hospitalized for the first time. Subjects with history of repeated tonsil inflammation, allergic rhinitis, asthma, obesity, recent acute infection or other systemic diseases were excluded. The 50 children with OSAHS were diagnosed by lateral nasopharynx X-ray as adenoidal hypertrophy, out of whom 46 were more than grade II with bilateral adenoidal hypertrophy.

Meanwhile, 52 healthy, age- and gender-matched children without related or systemic diseases were enrolled as a control group, comprising 33 boys and 19 girls aged 77.13 months on average (36-143 months). Preoperative examinations (chest X-ray, electrocardiography, coagulation function, as well as routine hematological and biochemical tests) showed normal results.

This was a prospective study, so a detailed program had been designed before the study started. All enrolled children had identical life, diet and living environment in hospital.

### Methods

The peripheral venous blood was collected and added in a tube containing ethylene diamine tetra-acetic acid for T cell subset analysis using flow cytometry (FACSCalibur, BD, USA). Blood samples in another two anticoagulant tubes were left still. The supernatant in one tube was used to detect cytokines by flow cytometry, and that in the other tube was employed to detect immunoglobulins and complements by a Siemens BN II immunoassay system (Germany).

### Statistical analysis

All data were analyzed by SPSS version 16.0. The categorical data that were identified by the Kolmogorov-Smimov test as approximately normally distributed were expressed as mean ± standard deviation (X ± SD). Inter-group comparisons were performed by the t test or analysis of variance.

## RESULTS

### Baseline clinical data

The Chi-square test revealed no significant difference between the gender ratio of the two groups (P>0.05), and the rank sum test showed the two groups had similar age ranges (P>0.05). The baseline clinical data of the two groups are summarized in Table-I.

**Table-I:** Baseline clinical data.

Group	Number of cases	Boy	Girl	Average age (month)
OSAHS	50	40 (80.0%)	10 (20.0%)	79.38
Control	52	33 (63.5%)	19 (36.5%)	77.13
P		>0.05	>0.05	>0.05

### T cell subsets

**Fig. 1:**
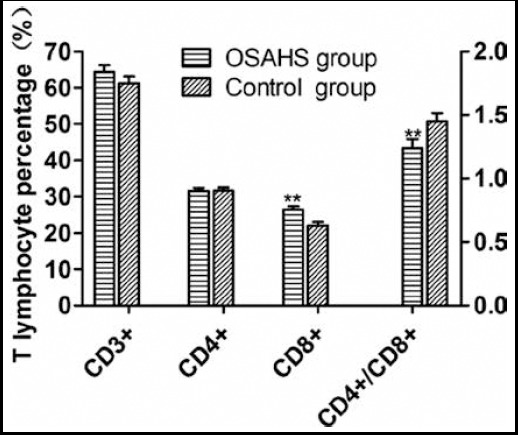
T cell subset analysis results. Compared with control group, *P<0.05, **P<0.01.

T cell subset analysis showed that the percentage of CD8^+^ T lymphocytes in children with OSAHS was (26.47 ± 1.52)% which significantly exceeded that of control group ((21.94 ± 1.92)%) (P<0.05). The two groups had similar percentages of CD3^+^ ((64.47 ± 3.20)% vs. (64.48 ± 3.10)%) and CD4^+^ T lymphocytes ((31.51 ± 1.48)% vs. (31.62 ± 1.52)%) (P>0.05). The OSAHS group had a significantly lower CD4^+^/CD8^+^ ratio (1.24 ± 0.12) than that of the control group (1.45 ± 0.11) (P<0.05) (Fig.1).

### Serum cytokine levels

**Fig. 2:**
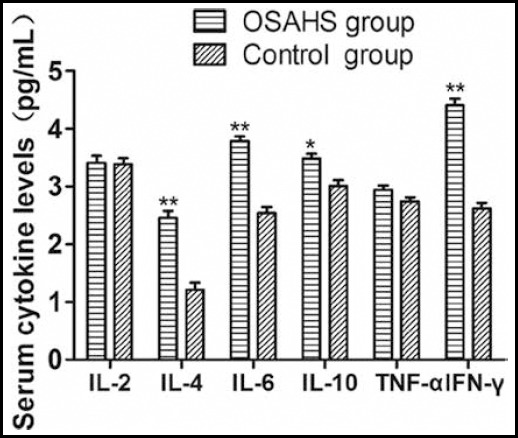
Serum cytokine levels. Compared with control group, *P<0.05, **P<0.01.

Compared with the control group, the OSAHS group had significantly higher serum IL-4 level ((2.45 ± 0.21) pg/ml vs. (1.21 ± 0.22) pg/ml) (P<0.05), IL-6 level ((3.75 ± 0.19) pg/ml vs. (2.54 ± 0.17) pg/ml) (P<0.05), IL-10 level ((3.45 ± 0.19) pg/ml vs. (3.01 ± 0.17) pg/ml) (P<0.05) and IFN-γ level ((4.41 ± 0.19) pg/ml vs. (2.61 ± 0.19) pg/ml) (P<0.05). However, their IL-2 ((3.41 ± 0.21) pg/ml vs. (3.38 ± 0.19) pg/ml) and TNF-α levels ((3.01 ± 0.21) pg/ml vs. (2.89 ± 0.23) pg/ml) were similar (P>0.05) (Fig.2).

### Immunoglobulin and complement levels

**Fig. 3:**
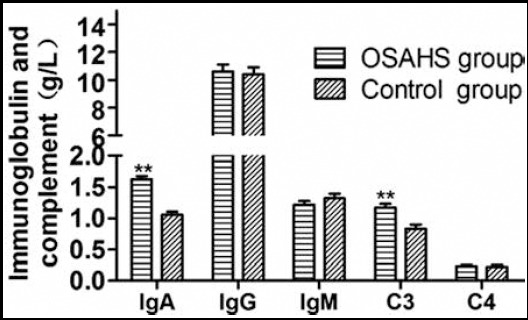
Immunoglobulin and complement levels. Compared with control group, *P<0.05, **P<0.01.

The serum IgA ((1.63 ± 0.09) g/l vs. (1.03 ± 0.12) g/l) and C3 levels ((1.17 ± 0.11) g/l vs. (0.83 ± 0.12) g/l) of the OSAHS group significantly exceeded those of the control group (P<0.05), but their IgG ((10.63 ± 0.89) g/l vs. (10.43 ± 0.91) g/l), IgM ((1.22 ± 0.11) g/l vs. (1.32 ± 0.13) gl) and C4 levels ((0.23 ± 0.02) g/l vs. (0.18 ± 0.02) g/l) were similar (P>0.05) (Fig.3).

## DISCUSSION

Lymphocytes are the most important cell populations in the immune system, reflecting the immune status of human body. Upon immune response, lymphocytes in the peripheral blood develop and differentiate into subsets with various functions.^5^ Obstruction of the upper airway in children with OSAHS affects their normal sleep, rest and neuroendocrine regulation, so they become susceptible to other diseases due to cell immune disorders. Abnormal numbers and functions of the subsets are bound to induce pathological changes by leading to immune disorders.^6^ The levels and proportions of lymphocyte subsets in normal human body are well maintained, and they interact mutually to keep immune functions normal. T helper lymphocytes and inhibitory lymphocytes play central roles in immune regulation. Generally, increase in CD4^+^/CD8^+^ ratio indicates an enhanced cell immune function, whereas decrease in the ratio suggests a weakened function.^7^ By detecting the lymphocyte subsets in peripheral blood, Qin et al. found that adult patients with moderate or severe OSAHS had significantly lower percentages of CD4^+^ T lymphocytes but significantly higher percentages of CD8^+^ T lymphocytes than those of the control group. Their CD4^+^/CD8^+^ ratios were thus significantly lower. CD8^+^ T lymphocytes were closely related with the atherosclerosis of adult patients.^8^ Similarly, in this study, the OSAHS group had a significantly higher percentage of CD8^+^ T lymphocytes (P<0.05) and a significantly lower CD4^+^/CD8^+^ ratio (P<0.05) than those of the control group, suggesting that children with OSAHS underwent immune function decline.

Cells in adenoids and tonsils contain IgM, IgA and IgG, commonly as the first defense line against respiratory tract infections.^9^ Khan et al. reported that the IgA and IgM levels of children with adenoidal hypertrophy surpassed those of normal ones. The serum IgM, IgA and IgG levels of children one month and six months after adenoidectomy and tonsillectomy were similar to those before surgeries.^10^ Likewise, Li et al. found that the serum IgM, IgA and IgG levels of adults with OSAHS were not significantly different from those of normal controls, and the preoperative and postoperative levels did not differ significantly either. However, there were two cases of deficiency in immunoglobulins, who were diagnosed only when repeated postoperative upper airway infection was not alleviated. Therefore, they recommended to perform immune function examination before tonsillectomy.^11^ The serum IgA level of children with OSAHS herein was significantly higher than that of the control group (P<0.05), being consistent with the finding of Khan et al.^10^ Children with OSAHS are subjected to a series of hypoxia-reoxygenation processes induced by repeated airway collapse during sleep, resembling the pathological and physiological processes of ischemia-reperfusion. Alternate hypoxia and reoxygenation processes evidently increase superoxide anions, so the numbers of neutrophils and monocytes skyrocket to induce a series of damages.

C3, as the center of two main activation pathways of complements, has crucial biological functions.^12^ The metabolites of C3 attack complexes and then autologous tissues and cells. Li et al. reported that in 178 adults with OSAHS, C3 markedly increased but IgM decreased upon humoral immune response. The C3 level was positively correlated with AHI but negatively correlated with percutaneous minimum blood oxygen saturation.^13^ In this study, the OSHAS group had a significantly higher serum C3 level than that of the control group, suggesting that OSAHS was a chronic, low-grade inflammatory disease.

Cytokines and mediators predominantly control the pathological and physiological processes of OSAHS induced by hypoxia at night and sleep disorders. Repeated hypoxemia and OSAHS-related awakening are both associated with oxidative stress and systemic inflammation, even if patients are not obese.^14^ After surgery, children with OSAHS have higher TNF-α and IL-6 levels in tonsil tissues than those of the children with repeated tonsillitis. Tam et al. reported that the OSAHS group (n=44, 7.3 years old on average) had significantly higher levels of INF-γ and IL-8 than those of the control group (n=69, 7.6 years old on average), but the two groups had similar IL-2, IL-4, IL-6, IL-8 and TNF-α levels.^15^ Serum IL-6 level increased but IL-10 level decreased in children with OSAHS, which were recovered to normal after adenoidectomy and tonsillectomy. Li et al. also found that the raised serum levels of IL-6, IL-8 and TNF-α in children with OSAHS were recovered to normal after treatment.^16^

IL-4 is an indispensable and versatile cytokine upon immune response. As the most potent IgE-regulating factor, IL-4 plays a key role in the onset and progression of allergic inflammation.^17^ IL-6 is an essential inflammatory cytokine that is involved in the upper airway infection and closely associated with the onset of allergic rhinitis and asthma. Vgontzas et al. found that IL-6 and TNF-α were related with lethargy and fatigue, and positively correlated with AHI, indicating that they were correlated with the sleep disorders of children with OSAHS.^18^ Ridker et al. reported that healthy subjects with elevated serum IL-6 levels were significantly more prone to future myocardial infarction, so IL-6 was an independent risk factor for predicting cardiovascular events.^19^ IL-10 and INF-γ are secreted by Th1 cells. IL-10 can inhibit a variety of pro-inflammatory cytokines by promoting the secretion of other anti-inflammatory cytokines, inhibiting the aggregation of eosinophils in the airway, excluding them from inflammatory sites and suppressing IgE production, finally mitigating the airway inflammatory response. The functions of INF-γ mostly antagonize those of IL-4. By inhibiting the differentiation of Th2 cells, INF-γ can suppress IgE production in B cells. Similar outcomes of patients with asthma have been reported by Wong et al.^20^

## CONCLUSION

Children with OSAHS had significantly higher serum levels of IL-4, IL-6, IL-10 and INF-γ than those of the control group, but the two groups had similar IL-2 and TNF-α levels. As evidenced by the increase of IL-4 and IL-6 levels, children with OSAHS were in a systemic inflammatory status. Simultaneous elevation of IL-4, IL-10 and INF-γ levels may be related to autoimmune protection, and increased secretion of the latter two counteracted the pathogenic effects of IL-4.

## References

[1] Dyugovskaya L, Lavie P, Lavie L (2005). Lymphocyte activation as a possible measure of atherosclerotic risk in patients with sleep apnea. Ann N Y Acad Sci.

[2] Mingari MC, Ponte M, Bertone S, Schiavetti F, Vitale C, Bellomo R (1998). HLA class I-specific inhibitory receptors in human T lymphocytes: interleukin 15-induced expression of CD94/NKG2A in superantigen-or alloantigen-activated CD8+T cells. Proc Natl Acad Sci USA.

[3] Wieckowski EU, Visus C, Szajnik M, Szczepanski MJ, Storkus WJ, Whiteside TL (2009). Tumor-derived microvesicles promote regulatory T cell expansion and induce apoptosis in tumor-reactive activated CD8+T lymphocytes. J Immunol.

[4] Blanco P, Pitard V, Viallard JF, Taupin JL, Pellegrin JL, Moreau JF (2005). Increase in activated CD8+T lymphocytes expressing perforin and granzyme B correlates with disease activity in patients with systemic lupus erythematosus. Arthritis Rheum.

[5] Kyaw T, Winship A, Tay C, Kanellakis P, Hosseini H, Cao A (2013). Cytotoxic and proinflammatory CD8+T lymphocytes promote development of vulnerable atherosclerotic plaques in apoE-deficient mice. Circulation.

[6] Yu W, Jiang N, Ebert PJ, Kidd BA, Müller S, Lund PJ (2015). Clonal deletion prunes but does not eliminate self-specific αβCD8+T lymphocytes. Immunity.

[7] Renrick AN, Thounaojam MC, Dudimah DF, Thomas P, Pellom ST, Uzhachenko RV (2016). Bortezomib enhances expression of effector molecules in antitumor CD8+T lymphocytes by modulating Notch-NF-kB-miR-155 crosstalk. Cancer Res.

[8] Qin YH, Cai Z, Qiu YR (2010). T cell subsets and NK cell level in patients with obstructive sleep apnea/hypopnea syndrome. Guangdong Med J.

[9] Clavijo PE, Frauwirth KA (2012). Anergic CD8+T lymphocytes have impaired NF-κB activation with defects in p65 phosphorylation and acetylation. J Immunol.

[10] Khan MN, Pichichero ME (2013). CD4 T cell memory and antibody responses directed against the pneumococcal histidine triad proteins PhtD and PhtE following nasopharyngeal colonization and immunization and their role in protection against pneumococcal colonization in mice. Infect Immun.

[11] Li FJ, Yin MD (2005). Effects of tonsillectomy on serum immune globulins of 35 cases. J Qiqihar Uni Med.

[12] Soudja SMH, Ruiz AL, Marie JC, Lauvau G (2012). Inflammatory monocytes activate memory CD8+T and innate NK lymphocytes independent of cognate antigen during microbial pathogen invasion. Immunity.

[13] Li ZG, Li TP, Ye H, Feng Y, Li DQ (2011). Immune function changes in patients with obstructive sleep apnea hypopnea syndrome. J Southern Med Uni.

[14] Sukumar M, Liu J, Ji Y, Subramanian M, Crompton JG, Yu Z (2013). Inhibiting glycolytic metabolism enhances CD8+T cell memory and antitumor function. J Clin Invest.

[15] Tam CS, Wong M, McBain R, Bailey S, Waters KA (2006). Inflammatory measures in children with obstructive sleep apnoea. J Paediatr Child Health.

[16] Li AM, Lam HS, Chan MH, So HK, Ng SK, Chan IH (2008). Inflammatory cytokines and childhood obstructive sleep apnoea. Ann Acad Med Singapore.

[17] Johns MW (1993). Daytime sleepiness, snoring, and obstructive sleep apnea: the Epworth Sleepiness Scale. Chest.

[18] Vgontzas AN, Papanicolaou DA, Bixler EO, Hopper K, Lotsikas A, Lin HM (2000). Sleep apnea and daytime sleepiness and fatigue: relation to visceral obesity, insulin resistance, and hypercytokinemia. J Clin Endocrinol Metab.

[19] Ridker PM, Rifai N, Stampfer MJ, Hennekens CH (2000). Plasma concentration of interleukin-6 and the risk of future myocardial infarction among apparently healthy men. Circulation.

[20] Wong CK, Ho CY, Ko FW, Chan CH, Ho AS, Hui DS (2001). Proinflammatory cytokines (IL-17, IL-6, IL-18 and IL-12) and Th cytokines (IFN-gamma, IL-4, IL-10 and IL-13) in patients with allergic asthma. Clin Exp Immunol.

